# Роль государственного регистра сахарного диабета в оценке эпидемиологической ситуации в Кыргызстане и г. Бишкеке

**DOI:** 10.14341/probl13502

**Published:** 2025-05-20

**Authors:** Р. Б. Султаналиева, Н. К. Абылова, Б. З. Жунусова

**Affiliations:** Диабетическая и эндокринологическая ассоциация Кыргызстана; Кыргызский государственный медицинский институт переподготовки и повышения квалификации; Диабетическая и эндокринологическая ассоциация Кыргызстана; Кыргызский государственный медицинский институт переподготовки и повышения квалификации; Диабетическая и эндокринологическая ассоциация Кыргызстана; Кыргызский государственный медицинский институт переподготовки и повышения квалификации; Международная высшая школа медицины

**Keywords:** регистр, сахарный диабет, распространенность, заболеваемость, смертность, диабетические осложнения, диабет и беременность

## Abstract

**ОБОСНОВАНИЕ:**

ОБОСНОВАНИЕ. Государственный регистр больных сахарным диабетом (ГРСД) впервые внедрен на всей территории Киргизской Республики (КР) с 2015 г. и стал одним из приоритетных направлений в эндокринологической службе республики. Создание национального регистра стало существенным шагом на пути оптимизации помощи лицам с сахарным диабетом (СД). В настоящее время регистр в КР не работает на online-программном обеспечении, поэтому вся информация о СД оценивается статически, представляя собой одномоментный срез на период окончания календарного года.

**ЦЕЛЬ:**

ЦЕЛЬ. Изучить эпидемиологические аспекты (распространенность, заболеваемость, смертность), а также частоту осложнений СД в Кыргызстане и крупном населенном городе страны — Бишкеке.

**МАТЕРИАЛЫ И МЕТОДЫ:**

МАТЕРИАЛЫ И МЕТОДЫ. Объект исследования — база данных регистра СД по КР и г. Бишкеку (данные в динамике с 2016–2023 гг.).

**РЕЗУЛЬТАТЫ:**

РЕЗУЛЬТАТЫ. Общая численность пациентов с СД, состоящих на диспансерном учете в Кыргызстане на 01.01.2024 г., по данным ГРСД, составила 85 142 человека, что составило 1,2% от общего населения республики. В Кыргызстане, по данным регистра, среди больных сахарным диабетом 1 типа (СД1) доля лиц мужского пола составила 52,4%, а лиц женского пола — 47,6%, в группе пациентов с сахарным диабетом 2 типа (СД2) преобладали лица женского пола (59,9%). Распространенность СД в КР в динамике за анализируемый 8-летний период (2016–2023 гг.) среди пациентов с СД1 возросла с 37/100 тыс. населения до 49,8/100 тыс. населения (в 1,35 раза), а с СД2 — с 847,6/100 тыс. населения до 1159,0/100 тыс. населения (в 1,37 раза). Динамика ежегодной заболеваемости СД1 по КР составляет в среднем 3,6 на 100 тыс. населения, а СД2 — возросла в период с 2016 по 2019, увеличившись на 27,6%, с 85 до 108,5 на 100 тыс. населения, и снизилась до 94 на 100 тыс. населения в 2023 году. Наиболее распространенными осложнениями среди пациентов с СД1 по республике остаются: нейропатия, ретинопатия, нефропатия, а при СД2 — нейропатия, ретинопатия. По КР за анализируемый период наблюдается стабилизация и/или снижение частоты большинства диабетических осложнений, за исключением острых нарушений мозгового кровообращения (ОНМК), синдрома диабетической стопы (СДС), острого инфаркта миокарда (ОИМ).

**ЗАКЛЮЧЕНИЕ:**

ЗАКЛЮЧЕНИЕ. ГРСД в Кыргызстане за 8 лет работы в статическом режиме позволил провести клинико-эпидемиологический мониторинг, обеспечив наблюдение за пациентами с момента включения в регистр и предоставив данные о распространенности, заболеваемости и осложнениях СД. Однако работа регистра затруднена из-за отсутствия доступа к интернету и компьютерам в ряде регионов, а также по причине своевременного ввода данных. Перевод ГРСД в онлайн-формат необходим для эффективного мониторинга и контроля ключевых показателей заболевания в режиме реального времени.

## Abstract (en)

**BACKGROND:**

BACKGROND: The State Register of Diabetes Patients (SRDP) was first introduced throughout the Kyrgyz Republic in 2015 and has become one of the priority areas in the endocrinological service of the republic. The creation of a national registry was a significant step towards optimizing care for people with diabetes mellitus (DM). Currently, the register in the Kyrgyz Republic does not work online software, so all information about diabetes is assessed statically, representing a one-time snapshot for the end of the calendar year.

**AIM:**

AIM: To study epidemiological aspects (prevalence,incidence mortality), as well as the frequency of complications of diabetes mellitus in Kyrgyzstan and the largest populated city in the country - Bishkek.

**MATERIALS AND METHODS:**

MATERIALS AND METHODS: The object of the study is the database of the DM register for the Kyrgyz Republic and the city of Bishkek (data over time from 2016–2023).

**RESULTS:**

RESULTS: The total number of patients with diabetes registered in the dispensary in Kyrgyzstan as of 01/01/2024, according to the SRDP, was 85,142 people, which amounted to 1.2% of the total population of the republic. In Kyrgyzstan, according to the register, among patients with T1DM, the proportion of males was 52.4%, and females — 47.6%, and in the group of patients with T2DM, females predominated (59.9%). The prevalence of diabetes in the Kyrgyz Republic over the analyzed 8-year period (2016–2023) among patients with type 1 diabetes increased from 37/100ths of the population to 49.8/100ths of the population (1.35 times), and with T2DM — from 847.6/100ths population to 1159.0/100ths population (1.37 times). The dynamics of the annual incidence of T1DM in the Kyrgyz Republic averages 3.6 per 100ths population, and T2DM increased from 2016 to 2019, increasing by 27.6%, from 85 to 108.5/100ths population and decreased to 94/100 thousand population in 2023. The most common complications among patients with type 1 diabetes in the republic remain: neuropathy, retinopathy, nephropathy, and for type 2 diabetes — neuropathy, hypertension, retinopathy. According to the register, during the analyzed period there was a stabilization and/or decrease in the frequency of most diabetic complications, with the exception of stroke, diabetic foot (DF), and acute micardial infarction (AMI).

**CONCLUSION:**

CONCLUSION: The SRDP in Kyrgyzstan, over 8 years of operation in static mode, has allowed for clinical and epidemiological monitoring, ensuring observation of patients from the moment of inclusion in the registry and providing data on the prevalence, incidence and complications of diabetes. However, the registry’s operation is hampered by the lack of access to the Internet and computers in a number of regions, as well as the timeliness of data entry. The transfer of the SRDP to an online format is necessary for effective monitoring and control of key disease indicators in real time.

Сахарный диабет (СД) представляет собой глобальную эпидемию, взятую под контроль Организацией Объединенных Наций (ООН) и национальными системами здравоохранения по всему миру [1]. Согласно данным Международной федерации диабета, число пациентов с СД в возрасте 20–79 лет в мире достигло 537 млн, а к 2045 г. прогнозируется практически двукратное увеличение до 783 млн человек (на 46%) [2], а к 2050-му ожидается, что более 1,31 млрд человек по всему миру будут жить с диабетом [3].

Неуклонное увеличение случаев СД, системные осложнения, ранняя инвалидизация, высокая смертность от сердечно-сосудистых заболеваний и значительное финансовое бремя для людей и системы здравоохранения привели к принятию в 2013 г. Глобального плана действий по борьбе с диабетом и другими неинфекционными заболеваниями [4], который должен сопровождаться системой мониторинга, включающей ряд индикаторов для отслеживания прогресса. Важность создания регистра и информационных систем по диабету была отмечена в связи с существенным увеличением числа пациентов и необходимостью получения достоверных данных о новых случаях заболевания, наличии осложнений, результатах лабораторных исследований и обеспечении эффективного контроля и мониторинга состояния здоровья пациентов.

Из 53 стран Европейского региона ВОЗ семь имеют национальные регистры диабета, 21 имеет специальный регистр диабета для пациентов определенного возраста или типа диабета, а 13 не имеют национального реестра. К странам, имеющим регистр, относится и Киргизская Республика (КР), но отмечено, что «национальный реестр предназначен для включения людей с диагностированным диабетом, но он неполный. В настоящее время все амбулаторные обращения фиксируются в клинической информационной системе» [5].

Государственный регистр больных СД (ГРСД), впервые внедренный на всей территории КР с 2015 г., стал одним из приоритетных направлений в эндокринологической службе республики. В настоящее время регистр в Киргизской Республике не использует онлайн-программное обеспечение, поэтому вся информация о сахарном диабете оценивается статически, представляя собой одномоментный срез на конец календарного года.

## ЦЕЛЬ

Изучить эпидемиологические аспекты СД, такие как распространенность, заболеваемость и смертность, а также частоту осложнений СД в Кыргызстане и в столице — городе Бишкеке.

## МАТЕРИАЛЫ И МЕТОДЫ

Объект исследования — база данных регистра СД по КР и г. Бишкеку на 01.01.2024 г. Показатели распространенности, заболеваемости и смертности среди пациентов с сахарным диабетом 1 и 2 типов (СД1 и СД2), а также частота диабетических осложнений проанализированы в динамике за период с 2016 по 2023 гг. Для расчета этих показателей использовались данные о численности населения Киргизской Республики и города Бишкека по годам, и рассчитывались на 100 тысяч человек. Частота осложнений определялась как процент пациентов с соответствующим осложнением от общего числа зарегистрированных в регистре случаев СД за период с 2016 по 2023 гг.

## Этическая экспертиза

Протокол исследования №4 от 16 сентября 2022 г. был одобрен этическим комитетом при Кыргызско-Российском Славянском университете.

## РЕЗУЛЬТАТЫ

По данным ГРСД на 01.01.2024 г., общая численность пациентов с диабетом, состоящих на диспансерном учете в Кыргызстане, достигла 85 142 человек, что составило 1,2% от общего числа жителей республики. В Бишкеке на диспансерном учете в государственных учреждениях находится 18 460 пациентов с диабетом, что составило 1,5% населения города (табл. 1).

**Table:** Таблица 1. Общая численность пациентов с сахарным диабетом в Кыргызстане и г. Бишкеке на 01.01.2024 г.

	Кыргызстан	Бишкек
СД1	СД2	СД1	СД2
Дети	799	-	243	
Подростки	332	-	84	
Взрослые	2375	81 635	910	17 223
Всего	3506	81 635	1237	17 223
Всего на 01.01 2024	85 141	18 460 человек(21,7% от всех пациентов с СД в КР)

В последние годы значительное внимание уделяется гендерным различиям заболеваемости СД. По данным Международной федерации диабета (IDF) от 2021 г., распространенность СД2 в возрастной категории молодых и лиц среднего возраста выше среди мужчин, чем среди женщин. СД2 у мужчин обычно проявляется в более раннем возрасте и при меньшем индексе массы тела (ИМТ) по сравнению с женщинами [2]. Однако с возрастом распространенность заболевания у женщин начинает значительно возрастать и превышает таковую у мужчин, что связывают с более распространенной избыточной массой тела и возникающими гормональными изменениями, особенно в период менопаузы и после нее.

В Кыргызстане, по данным регистра, среди пациентов СД1 доля лиц мужского пола составила 52,4%, а лиц женского пола — 47,6%. А в группе пациентов с СД2 преобладали лица женского пола (59,9%). По г. Бишкеку и в случае СД1, и в случае СД2 преобладали пациенты женского пола (СД1 имеют 54,2%, СД2 — 61,6%).

## Распространенность СД в Кыргызстане и г. Бишкеке

Распространенность — показатель, оценивающий количество всех случаев заболевания, зарегистрированных в текущем календарном году, рассчитывается на 100 тыс. населения соответствующей возрастной группы. На рисунке 1 представлена динамика распространенности диабета в Кыргызстане в период с 2016 по 2023 гг.

**Figure:**
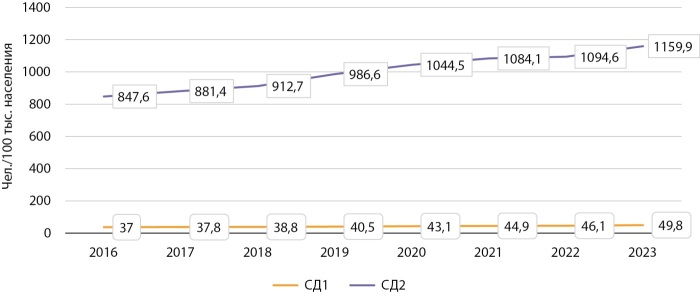
Рисунок 1. Динамика распространенности СД по Кыргызстану в период 2016–2023 гг. (на 100 тыс. населения).

Как видно из диаграммы, в КР наблюдается стабильный рост распространенности СД1 и СД2. За анализируемый 8-летний период показатель распространенности СД1 увеличился с 37/100 тыс. населения до 49,8/100 тыс. (в 1,35 раза), тогда как распространенность СД2 возросла с 847,6/100 тыс. населения до 1159/100 тыс. (в 1,37 раза).

Как и во всех странах мира, преимущественно увеличение распространенности СД отмечается за счет СД2. Общий темп прироста больных СД2 по КР за период 2016–2023 гг. составил 36,8%. Другие типы СД в регистре КР не представлены, хотя при анализе информационных карт, которые заполняются в учреждениях первичной медико-санитарной помощи (ПМСП) на каждого пациента, их количество доходило до 2,0%. Похоже, что при заполнении ГРСД они были включены в состав СД2. Стоит отметить, что истинная численность лиц с СД в КР в 3–4 раза превышает официально зарегистрированную и составляет, по данным IDF, примерно 256 400 человек [2]. Одной из основных проблем, связанных с ранней диагностикой, является бессимптомное течение на начальных стадиях болезни, время от возникновения заболевания до его выявления может достигать 7–12 лет.

В ГРСД по г. Бишкеку включены пациенты СД, состоящие на учете в десяти центрах семейной медицины (ЦСМ). Аналогично общему тренду по всей республике, в Бишкеке также наблюдается стабильный рост распространенности СД1 и СД2. За анализируемый 8-летний период показатель распространенности СД1 увеличился с 76/100 тыс. населения до 108/100 тыс. (в 1,42 раза), а СД2 — с 1178/100 тыс. населения до 1504/100 тыс. населения (в 1,28 раза). На рисунке 2 представлена динамика распространенности СД в г. Бишкеке.

**Figure:**
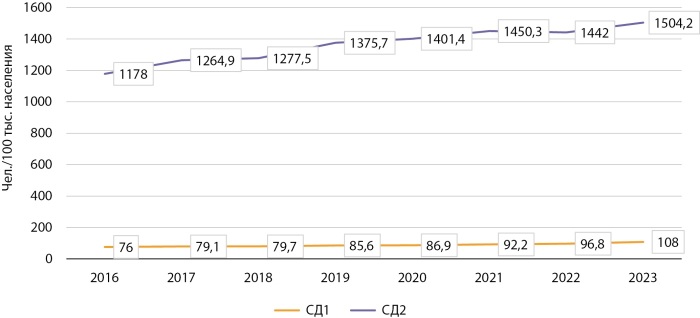
Рисунок 2. Динамика распространенности СД в г. Бишкеке в период 2016–2023 г. (на 100 тыс. человек).

Как видно из рисунка 2, подъем заболеваемости СД1 в г. Бишкеке пришелся на 2023 г. с темпом прироста 11,5%, тогда как общий прирост больных СД1 в столице за период 2016–2023 гг. составил 41,9%, что существенно выше аналогичного показателя по всей КР (34,5%). Темп прироста СД2 по г. Бишкеку за 8-летний период составил 27,6% против 36,8% по КР. На разницу вышеуказанных показателей по СД2 в стране и по г. Бишкеку, судя по всему, влияют профилактические факторы. Так, количество образовательных программ, семинаров, лекций и просвещающих мероприятий по профилактике СД, как среди врачей, так и населения, выше в г. Бишкеке. В группах риска (лица с ожирением, преддиабетом, артериальной гипертензией, дислипидемией и др.) превентивные мероприятия по профилактике нарушений углеводного обмена более активно проводятся в центрах семейной медицины (ЦСМ) г. Бишкеке по сравнению с регионами КР.

## Заболеваемость СД в Кыргызстане и г. Бишкеке

Заболеваемость (первичная, по обращаемости) — показатель, оценивающий количество новых случаев заболевания, впервые зарегистрированных в текущем календарном году. Динамика ежегодной заболеваемости СД1 по КР имеет стабильный характер и составляет в среднем 3,6 на 100 тыс. населения. Заболеваемость СД2 по всей республике имела тенденцию к росту в период с 2016 по 2019 гг., увеличившись на 27,6%, с 85 до 108,5 на 100 тыс. населения. После резкого скачка заболеваемости в 2019 году отмечается снижение регистрации первичной заболеваемости в последующие годы до 89,7 на 100 тыс., что, вероятно, связано с изменением приоритетов в пользу борьбы с коронавирусной инфекцией и снижением внимания к выявлению СД. И только с 2023 г. наметился восходящий тренд (рис. 3).

**Figure:**
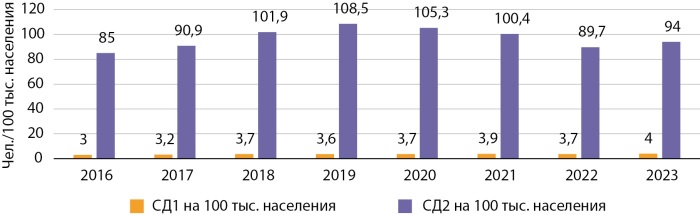
Рисунок 3. Динамика заболеваемости СД по Кыргызстану в 2016–2023 гг.

Ежегодная заболеваемость СД1 в столице в среднем составляет 5,4 на 100 тыс. населения, что выше такового показателя по КР в 1,5 раза. В г. Бишкеке новые случаи СД с 2016 по 2023 гг. были зарегистрированы у 8830 человек, из них СД1 выявлен у 469 (5,3%), из которых 50,7% случаев — дети. СД2 ежегодно в столице выявляется в среднем у 900–1000 человек. Следует отметить, что заболеваемость СД2 в Бишкеке (средний показатель — 100,2 на 100 тыс. населения за 8-летний период) сопоставима с таким же показателем по КР (102,8 на 100 тыс. населения за аналогичный период) (рис. 4). Новые случаи СД2 с 2016 по 2023 гг. были зарегистрированы у 8361 человека.

**Figure:**
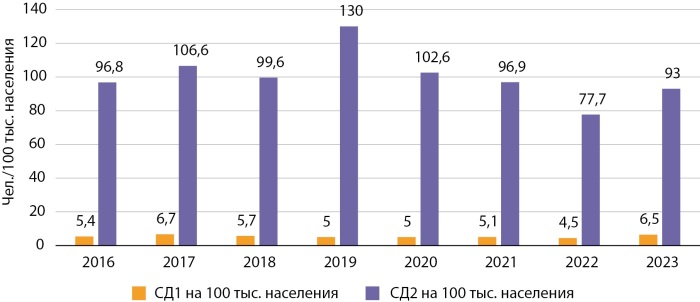
Рисунок 4. Заболеваемость СД в г. Бишкеке в динамике 2016–2023 гг.

## Сахарный диабет у детей и подростков Кыргызстана и г. Бишкека

Категория «дети» включает лица в возрасте до 15 лет, тогда как в категорию «подростки» отнесены лица в возрасте от 15 лет до 18 лет. В ГРСД Кыргызстана представлены данные о СД среди детей и подростков только по СД1. На 01.01.2024 г. в КР численность детей и подростков с СД1 составила 1131 человек, из которых 799 — это дети и 332 — подростки, а в г. Бишкеке — 327 случаев СД1 среди детей и подростков, из которых 243 — дети и 84 — подростки. Динамика распространенности СД1 у детей и подростков за 2016–2023 гг. представлена на рисунках 5 и 6.

**Figure:**
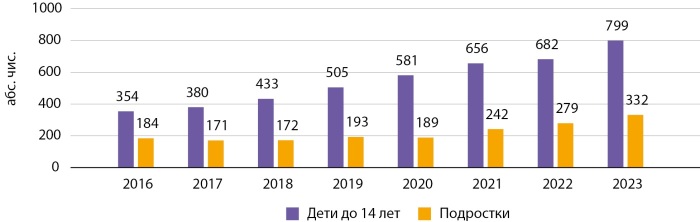
Рисунок 5. Динамика распространенности СД1 у детей и подростков в КР в динамике за 2016–2023 гг. (абс. чис.).

**Figure:**
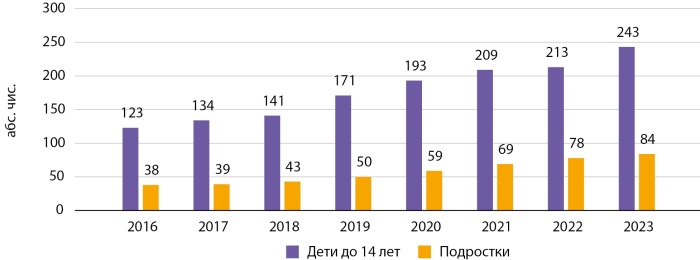
Рисунок 6. Динамика распространенности СД1 у детей и подростков в Бишкеке за период 2016–2023 гг. (абс. чис.).

Как видно из рисунков 5 и 6, с 2016–2023 гг. отмечался рост распространенности СД1 у лиц вышеуказанных групп, что согласуется с мировыми данными. По данным IDF, заболеваемость СД1 растет с каждым годом, и ежегодно заболевает более 108 тыс. детей в возрасте от 0 до 14 лет и более 41 тыс. подростков в возрасте от 15 до 19 лет [2]. СД1 является многофакторным заболеванием, к росту которого приводят различные механизмы взаимодействия генетической предрасположенности, факторов окружающей среды, состояния иммунной системы и др.

Сахарный диабет 2 типа, безусловно, встречается среди детей и подростков в КР. По данным мировых эпидемиологических исследований, заболеваемость СД2 у детей и подростков колеблется от 1 до 51 случая на 1000 детского населения, причем наиболее высокие уровни отмечаются среди национальных меньшинств, включая выходцев из Азии, Латинской Америки и коренных жителей США [2]. Статистические данные по СД2 у детей и подростков в КР не были представлены по ряду причин: популяционные эпидемиологические исследования и скрининг СД2 в данных возрастных группах в республике не проводился; СД2 у детей и подростков манифестирует в большинстве случаев с минимально выраженной симптоматикой или течет бессимптомно, что не ведет к ранней диагностике СД2; дифференциально-диагностический подход при явном диабете был не на должном уровне, поэтому все случаи диабета у детей и подростков расценивались как СД1.

## Сахарный диабет среди беременных женщин КР

В течение последних десятилетий наблюдается значительный рост заболеваемости СД среди женщин репродуктивного возраста [2, 6]. Согласно данным IDF за 2021 год, 21,1 млн беременных женщин (16,7%) страдали от различных форм гипергликемии во время беременности. Из них 80,3% составили женщины с гестационным сахарным диабетом (ГСД), 10,6% — женщины с СД, выявленным до беременности, и 9,1% — женщины с диабетом (включая СД1 и СД2), впервые обнаруженным в период беременности.

Анализ распространенности СД у беременных женщин в КР не проводился. Появившаяся в 2021 г. графа «ГСД» в регистре чаще всего не заполняется и остается пустой. Так, за 2023 г. в регистре по КР было обозначено всего 54 случая ГСД.

На официальном сайте национального статистического комитета КР (stat.kg) представлен отчет о заболеваниях женщин, осложнивших роды (5.02.00.26) в КР, в котором среди прочих заболеваний значится «сахарный диабет». По данному отчету СД осложнил беременность в 181 случае в 2022 г., что в пересчете на общее количество беременных за 2022 г. по КР составило всего 0,18% (табл. 2).

**Table:** Таблица 2. Заболевания женщин, осложнившие роды (в том числе сахарный диабет)

Наименование показателей	2015	2016	2017	2018	2019	2020	2021	2022
Анемия	86 810	91 933	84 410	83 156	82 597	80 103	77 617	69 857
Болезни системы кровообращения	667	695	691	798	859	665	640	730
Сахарный диабет	54	70	65	98	107	111	133	181
Поздний токсикоз	10 913	11 092	11 572	12 820	13 163	12 213	12 632	12 480
Болезни мочеполовой системы	3294	2215	2188	2293	2134	2232	1946	2050
Венозные осложнения	2467	3526	3977	4504	4434	4310	5178	5769

Однозначно, что данный отчет по СД у беременных женщин неполноценен и не отображает реальной картины распространенности и заболеваемости СД во время беременности. В связи с этим в КР были созданы клинические руководства и протоколы по диагностике и лечению ГСД и прегестационного диабета (ПГСД) для врачей всех специальностей, в соответствии с новыми данными и рекомендациями на основе доказательной медицины. И с 2024 г. в ГРСД все врачи будут обязаны заносить информацию о СД при беременности в государственный регистр.

## Смертность при сахарном диабете

Как известно, увеличение заболеваемости СД, соответственно, сопровождается и ростом смертности. СД по-прежнему занимает четвертое место среди причин смерти по всему миру. Доля смертей, связанных с диабетом, служит показателем относительного бремени этого заболевания в каждом регионе IDF [2]. Сообщается, что у пациентов с диабетом риск преждевременной смерти повышен на 15%, а ожидаемая продолжительность жизни сокращена примерно на 10 лет для пациентов с СД1 и на 20 лет для пациентов с СД2.

Показатели смертности пациентов с СД1 и СД2 в динамике по КР и г. Бишкеку представлены в таблице 3.

**Table:** Таблица 3. Показатели смертности пациентов с сахарным диабетом 1 и 2 типов в динамике по Кыргызстану и г. Бишкеку (на 100 тыс. населения)

	2016	2017	2018	2019	2020	2021	2022	2023
КР	6,7	6,8	7,3	7,0	9,8	7,1	6,2	6,7
г. Бишкек	5,0	4,3	3,4	2,8	4,7	2,2	3,6	2,7

Как видно из таблицы, в КР за анализируемый 8-летний период отмечается небольшое поступательное повышение показателя смертности до 9,8 на 100 тыс. населения к 2020 г., далее постепенно снижается до 6,7. По г. Бишкеку наблюдается снижение с 5,0 на 100 тыс. населения до 2,7 на 100 тыс. населения [7]. Эти данные, безусловно, являются очень низкими, не отражают реальных показателей смертности, связанных с СД, что связано с проблемами регистрации смерти. Как известно, более 50–60% больных СД страдают сердечно-сосудистыми заболеваниями (хроническая сердечно-сосудистая недостаточность (ХСН), инфаркт миокарда (ИМ), нарушения мозгового кровообращения (НМК) и другие острые сердечно-сосудистые события), более 30% — хронической болезнью почек (ХБП) с исходом в терминальную стадию почечной недостаточности, а также с различными инфекционными осложнениями. При летальном исходе в таких случаях лиц с СД не относят к группе больных, умерших от СД, хотя причина изменений сосудов и других осложнений могла быть обусловлена именно наличием СД.

Недостоверность этих показателей видно даже при сравнении с подобными данными соседних республик. Так, показатель смертности от СД в Казахстане колеблется от 34,55 до 114,31 на 100 тыс. населения, в Российской Федерации — от 88,4 до 142,2 на 100 тыс. населения, а в большинстве регионах мира доля смертей, связанных с диабетом, составляет более 5% от всех смертей [2][8]. Такие высокие показатели смертности среди больных СД объясняются развитием осложнений диабета. Так, 50–75% случаев смерти больных СД2 связаны с наличием макроангиопатий и в первую очередь с ишемической болезнью сердца (ИБС). В то же время ведущей причиной смерти больных СД1 является хроническая почечная недостаточность как следствие диабетической нефропатии. Таким образом, ГРСД КР неполностью отражают реальный вклад СД в смертность населения, поэтому цифры летальности в КР и г. Бишкеке резко занижены.

## Распространенность осложнений при сахарном диабете в Кыргызстане и г. Бишкеке на 01.01.2024 г.

Длительный недостаточный контроль гипергликемии у пациентов с СД часто приводит к развитию множества осложнений, преимущественно связанных с поражением мелких и крупных кровеносных сосудов (микро- и макроангиопатии). Самыми опасными являются нефропатия, ретинопатия, поражение сосудов сердца, головного мозга, периферических сосудов нижних конечностей. Именно они являются основной причиной инвалидизации и смертности больных.

Особое положение среди хронических осложнений занимает диабетическая полинейропатия (ДП) нижних конечностей, которая выявлена у 44% лиц с СД1 и у 50,4% лиц с СД2. Именно ДП является причиной синдрома диабетической стопы (СДС) у 11,5% лиц с СД1 и у 4,5% с СД2, которая привела к ампутации нижних конечностей у 0,5% и 0,8% лиц с СД1 и СД2. Диабетическая нефропатия, представляющая собой гломерулярный склероз и фиброз, обусловленные метаболическими и гемодинамическими изменениями при СД, выявляется у 17,2% пациентов с СД1 и у 8,5% пациентов с СД2. Однако ввиду отсутствия возможности расчета СКФ в ГРСД и ограничений в контроле уровня креатинина, связанных с недостаточной оснащенностью лабораторий в отдаленных регионах КР на уровне ПМСП, фактические показатели диабетической нефропатии могут быть недооценены. Диабети́ческая ретинопати́я (ДР) — поражение сетчатки глаза диабетического происхождения, выявлена, соответственно, у 29,9% и 23,9% лиц с СД1 и СД2. При отсутствии лечения данное осложнение приводит к полной слепоте. В отношении макрососудистых осложнений отмечена относительно меньшая частота: при СД1/СД2 частота ИБС 3,3%/13,7%, ИМ — 0,5%/1,6%, ОНМК 0,3/2,4% (рис. 7), а сопутствующая артериальная гипертензия (АГ) встречалась у 12,2/35,5% среди лиц с СД1 и СД2 соответственно.

**Figure:**
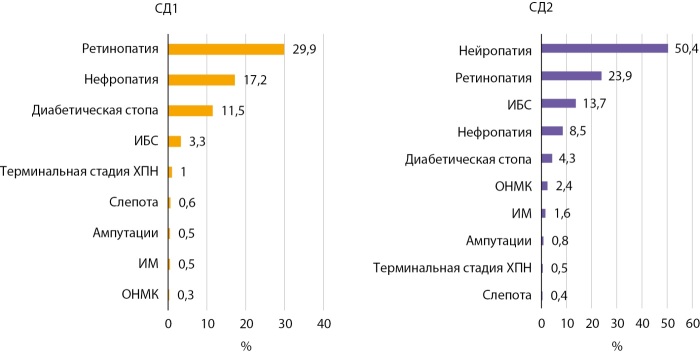
Рисунок 7. Распределение частоты осложнений при СД 1 и 2 типов в Кыргызстане на 01.01.2024 г.

Частота осложнений по г. Бишкеку представлена следующим образом: диабетическая нейропатия — 30% и 46,1%, диабетическая нефропатия, ХБП — 14,8 и 8,6%, ДР — 14,6 и 13,4%, ампутации стоп — 0,5 и 1% при СД1 и СД2 соответственно. В отношении макрососудистых осложнений отмечена относительно меньшая частота: при СД1/СД2 частота ИБС — 2,1%/20,7%, ИМ — 0,7%/3,7%, ОНМК — 0,1/4,2% (рис. 8). Коморбидная АГ среди пациентов с СД2 по г. Бишкеку встречалась чаще (46,2%) по сравнению с таковым показателем по КР (35,5%), что, вероятно, связано с лучшей выявляемостью и различиями в образе жизни.

**Figure:**
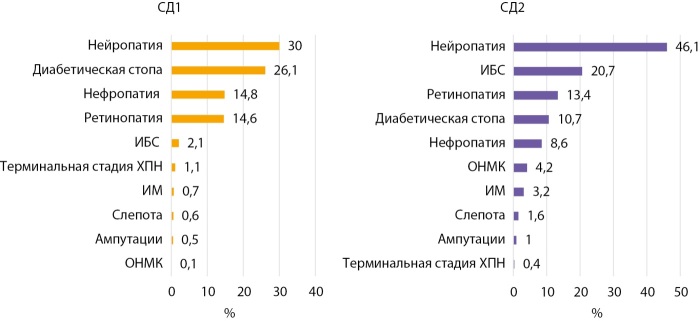
Рисунок 8. Распределение частоты осложнений при СД 1 и 2 типов в г. Бишкеке на 01.01.2024 г.

## Динамика основных диабетических осложнений в КР в период 2016–2023 гг.

Динамика частоты основных диабетических осложнений в Кыргызстане и г. Бишкеке в период 2016–2023 гг. представлены на рисунках 9, 10, 11, 12.

**Figure:**
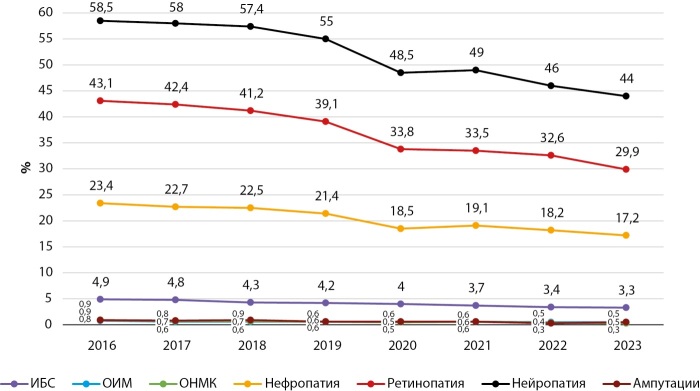
Рисунок 9. Динамика частоты осложнений при сахарном диабете 1 типа в Кыргызстане, 2016–2023 гг.

**Figure:**
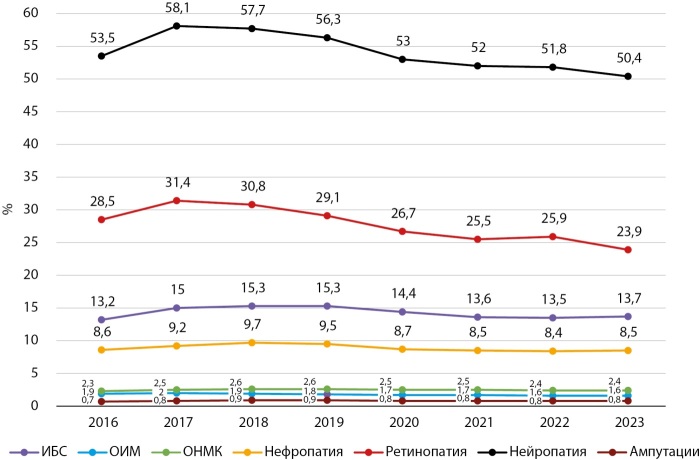
Рисунок 10. Динамика частоты осложнений при СД2 типа в КР, 2016–2023 гг.

**Figure:**
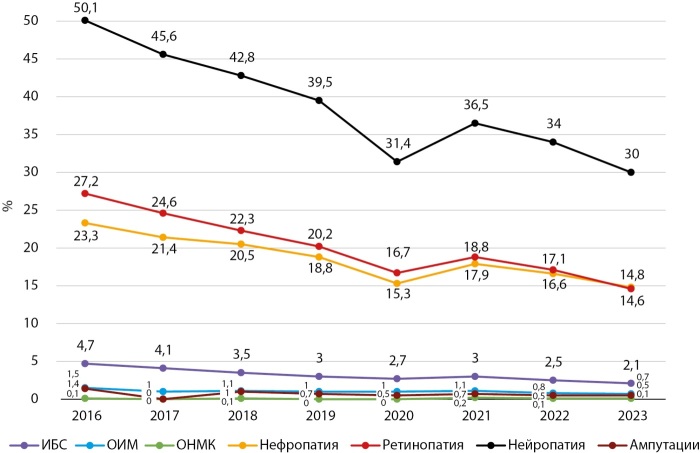
Рисунок 11. Динамика частоты осложнений СД1 в г. Бишкеке, 2016–2023 гг.

**Figure:**
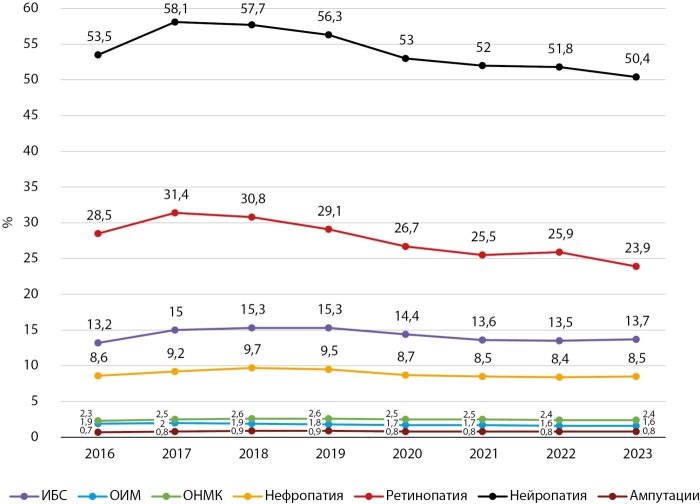
Рисунок 12. Динамика частоты осложнений СД2 в г. Бишкеке, 2016–2023 гг.

Как показывают представленные данные, в анализируемом периоде наблюдается стабилизация и/или снижение частоты большинства диабетических осложнений, за исключением ОНМК, СДС и ОИМ. Сосудистые заболевания головного мозга являются серьезной медико-социальной проблемой, так как занимают одно из ведущих мест в структуре общей смертности и инвалидности населения КР.

Снижение частоты диабетических осложнений получено в период реализации ряда национальных программ по здравоохранению в КР. В национальную программу реформирования системы здравоохранения КР «Ден соолук» (2011–2018 гг.) был заложен принцип ориентированности системы здравоохранения на потребности людей, усилен акцент на достижение конкретных результатов в улучшении показателей здоровья населения, а приоритетное внимание уделялось сердечно-сосудистым заболеваниям (ОИМ, инсульт и др.).

## ЗАКЛЮЧЕНИЕ

Государственный регистр сахарного диабета в Кыргызстане в течение 8 лет работал в статическом варианте. Немотря на это, он позволил провести клинико-эпидемиологический мониторинг СД в масштабах всей страны, дал возможность наблюдения за пациентами СД от момента включения в регистр на протяжении всего периода заболевания, получить систематическую достоверную информацию о рапространенности, зарегистрированной заболеваемости и осложнениях СД. Так, в течение анализируемого периода, как по всей республике, так и в столице, зафиксировано увеличение числа случаев СД на 100 тысяч населения во всех возрастных группах, что подчеркивает важность клинико-эпидемиологического мониторинга СД. Динамический анализ регистра показал, что СД представляет серьезную и глобальную проблему для Кыргызстана в силу роста и наличия различных осложнений, которые ложатся тяжелым бременем на здравоохранение страны. Однако работа ГРСД в офлайн-формате и ограничения в формировании отчетности существенно ограничивают эффективность его использования. В ряде регионов регистр сталкивается с дополнительными проблемами: отсутствием компьютеров или стабильного доступа к интернету, значительной нагрузкой на семейных врачей, которые зачастую не успевают своевременно вводить данные пациентов. Эти трудности препятствуют полному учету пациентов и своевременному анализу данных.

Для решения этих проблем и повышения эффективности клинико-эпидемиологического мониторинга необходимо перевести регистр в онлайн-формат. Это позволит проводить контроль и мониторинг ключевых показателей заболевания в режиме реального времени на уровне всей страны, что особенно важно для принятия своевременных медицинских и организационных решений.

## ДОПОЛНИТЕЛЬНАЯ ИНФОРМАЦИЯ

Финансирование работы. Работа выполнена по инициативе авторов без привлечения финансирования

Конфликт интересов. Авторы заявляют об отсутствии явных и потенциальных конфликтов интересов, связанных с публикацией настоящей статьи.

Участие авторов. Жунусова Б.З., Абылова Н.К. — анализ и интерпретация результатов исследования, написание текста статьи; Султаналиева Р.Б. — финальный анализ результатов и редактирование текста рукописи. Все авторы утвердили окончательную версию статьи перед публикацией и взяли на себя ответственность за все аспекты работы.

Благодарности. Всем медицинским специалистам (врачам, медицинским сестрам), ведущим активную работу по заполнению регистра СД.
